# Prophylactic subtotal colectomy in a patient with an AXIN2 mutation

**DOI:** 10.1093/jscr/rjab470

**Published:** 2021-11-29

**Authors:** Benjamin M Vierra

**Affiliations:** Harvard Medical School, Boston, MA, USA

## Abstract

A number of genetic syndromes that predispose patients to colon cancer have been well described, allowing for improved surveillance, diagnosis and treatment. However, other syndromes likely exist but have yet to be thoroughly characterized. I report the case of a 50-year-old woman who was found to have over 50 polyps on routine screening colonoscopy. Genetic sequencing revealed a mutation in Axin2, a rare gene implicated in oligodontia-colorectal cancer syndrome. It was later found that the patient lacks several permanent teeth, as does her mother, who also had a history of multiple colonic polyps. Given the rarity of Axin2 mutations, there are no guidelines for management of such patients. This highlights the importance of a thorough review of system when screening for colon cancer as well as documenting cases of Axin2 mutations to create management guidelines for these patients.

## INTRODUCTION

The Wnt/beta-catenin signaling pathway is a molecular pathway implicated in morphogenesis, embryonic pattern formation and oncogenesis. Central to the cascade is Wnt signal stabilization of beta-catenin, which regulates transcription factors to influence gene expression. In the absence of Wnt signaling, beta-catenin is phosphorylated and degraded by a multiprotein complex consisting of Axin (or its ortholog Axin2), glycogen synthase kinase 3 beta and adenomatous polyposis coli (APC). Axin2 thus inhibits beta-catenin signaling [1, 2]. Alterations in this pathway can increase the risk of developing malignancy or developmental issues. Mutations in APC, for instance, cause familial adenomatous polyposis. Although, rarer, Axin2 mutations, inherited in an autosomal dominant fashion, have been implicated in carcinogenesis and morphogenetic abnormalities. Specifically, they are associated with hypodontia and oligodontia, defined as the developmental lack of one or more, or six or more, permanent teeth, respectively. Axin2 mutations are also linked to malignancy, most notably colorectal cancer, due to its detection in a Finnish family in which several members suffered from tooth agenesis and colonic neoplasia [3]. Axin2 is thus a risk factor for oligodontia-colorectal cancer syndrome, predisposing patients to colorectal cancer and oligodontia. Understanding the association between dental health and colorectal cancer may be critical for screening, but to date, Axin2 is relatively unknown and oligodontia-colorectal cancer syndrome has not been well described. I add to the paucity of literature by reporting the case of a patient, with oligodontia and numerous colonic polyps, who was found to have an Axin2 mutation.

## CASE PRESENTATION

**Figure 1:**
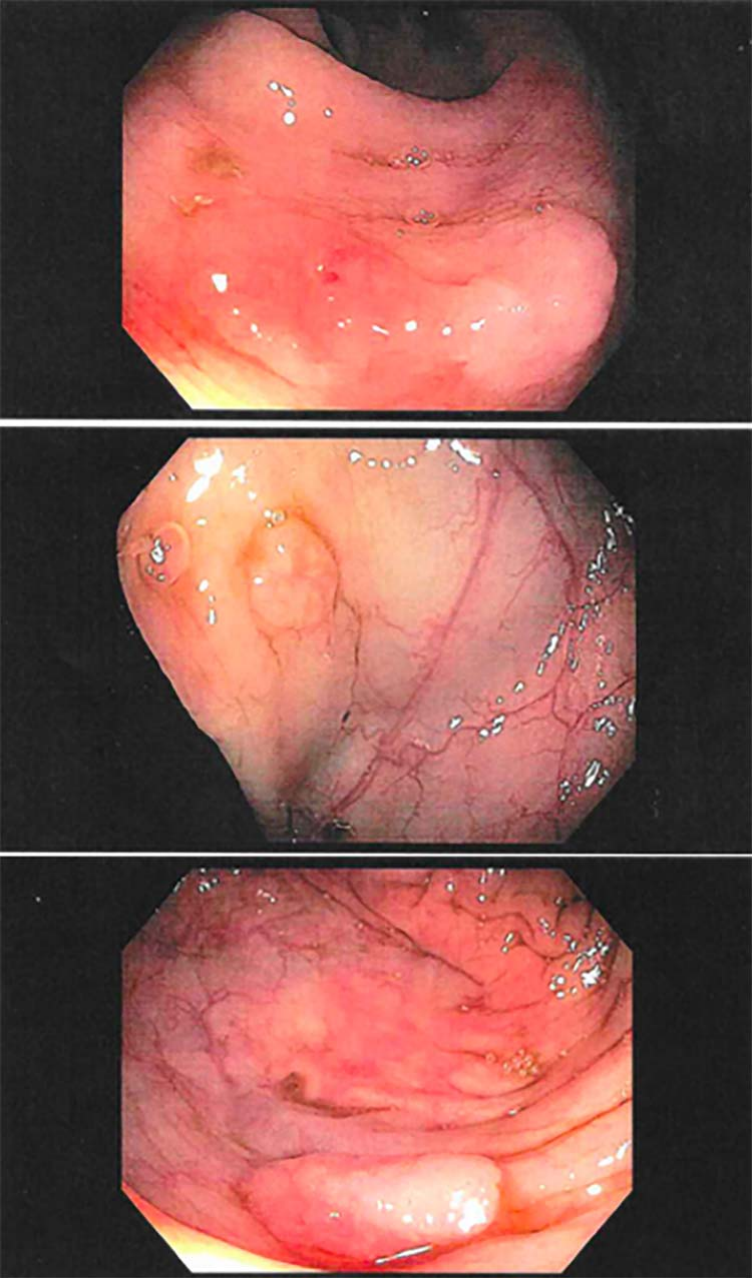
Examples of several polyps found on routine colonoscopy in the cecum (top, middle) and ascending colon (bottom).

A 50-year-old female, with a history of hypothyroidism and constipation and with a family history of a mother with multiple colonic polyps, presented for a routine screening colonoscopy. During the colonoscopy, dozens of polyps were identified throughout the colon. Many were removed, including 8 polyps between 3 and 10 mm from the cecum, 12 polyps between 3 and 10 mm from the ascending colon and one 6-mm polyp from the transverse colon (Fig. 1). Many residual polyps were left behind due to high burden of disease. Internal hemorrhoids and diverticulosis of the sigmoid colon were also noted. Of the 21 polyps removed, 20 were tubular adenomas and 1 was a lymphoid polyp. Given the unusual findings, the patient returned to clinic a few days later.

In clinic, a thorough history was performed. The patient’s only gastrointestinal history was chronic constipation, with bowel movements every day to every other day, sometimes requiring laxatives. She otherwise denied rectal bleeding, diarrhea or weight loss. She confirmed no family history of colon cancer, but her mother reportedly had many colon polyps. Her father passed away at an early age in a motor vehicle accident. None of her three healthy siblings or child had gastrointestinal issues. Given the high number of polyps in both the patient and mother, without significant personal or family history, she was referred for genetic testing.

Genetic testing returned positive for an AXIN2 mutation, which has been associated with oligodontia-colorectal syndrome. She again returned to clinic, where further investigation revealed that she lacked eight permanent teeth, requiring placement of a bridge and implants as a child. She recalled her mother having dental issues as well. She did not believe anyone else in her family had dental anomalies.

**Figure 2:**
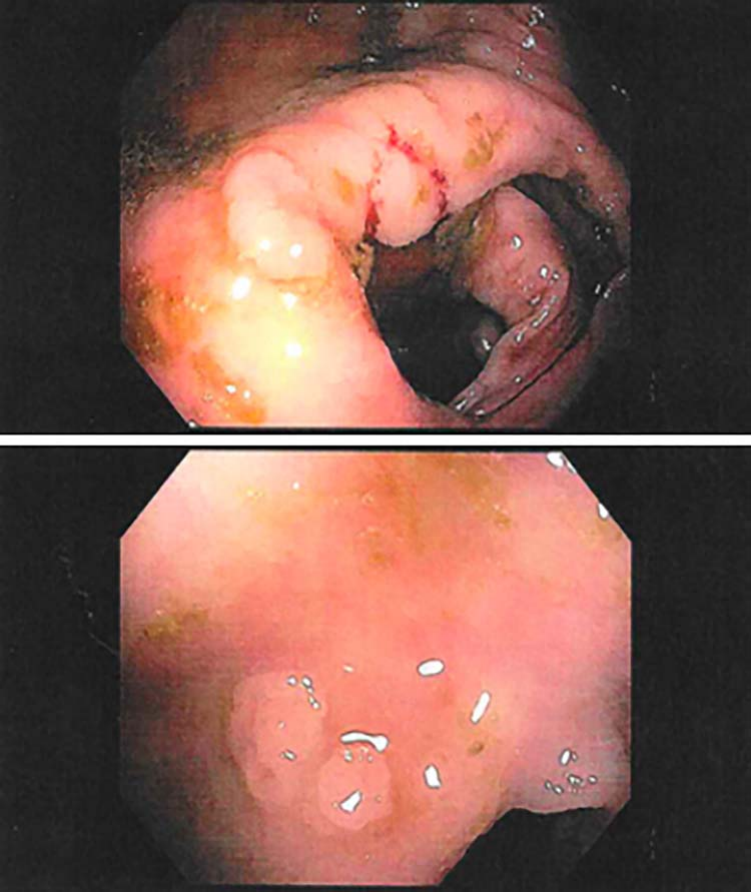
Examples of polyps found on follow-up colonoscopy in the ascending colon (top) and descending colon (bottom).

In addition to genetic counseling, she was recommended to undergo a follow-up colonoscopy. On repeat colonoscopy, 31 sessile polyps were found throughout the colon and were removed, still with multiple polyps left behind (Fig. 2).

Given the presence of an Axin2 mutation, her dental history and colonoscopy findings and her presentation not matching any known colorectal cancer syndrome, it was felt that she had or was at risk for oligodontia-colorectal cancer syndrome. However, there are no current diagnostic or management criteria. It was felt that, without intervention, she would likely need frequent surveillance colonoscopies and polypectomies to remove potentially precancerous polyps. Given this prospect, she was referred to a general surgeon and was ultimately elected for a subtotal colectomy with ileorectal anastomosis, as sparing the rectum would result in less bowel dysfunction. The rectum also did not have any polyps on previous colonoscopies and could easily be surveyed with sigmoidoscopies.

Following surgery, the patient had an unremarkable post-operative course and was discharged on post-operative day 3. One year following surgery, the patient had improved bowel function, with two to three bowel movements per day without the use of laxatives. A flexible sigmoidoscopy was negative for polyps in the rectum, with the plan to repeat a flexible sigmoidoscopy in 1 year and then to subsequently space out surveillance. To date, the family has not yet undergone genetic testing.

## DISCUSSION

Axin2 mutations are rarely described in existing literature. A frequently cited study comes from the observation of a Finnish family in which 11 members lacked 8 or more permanent teeth, and 8 of these patients had colorectal cancer or precancerous lesions; this was the first identification of Axin2 mutations as a risk factor for oligodontia-colorectal cancer syndrome [4]. Other studies have similarly found a link between Axin2 mutations and cancers, but to date, no large studies have been conducted. Therefore, the true risk of colorectal cancer in patients with an Axin2 mutation is unknown. There are thus no guidelines regarding the management of these patients. The patient described here felt that undergoing a prophylactic subtotal colectomy was preferable to repeated, frequent colonoscopies and polypectomies.

This case is important for several reasons. First, it could be argued that patients undergoing screening for colorectal cancer should be asked about dental health, especially if they are found to have unusual findings on colonoscopy. Second, although oligodontia-colorectal syndrome is rare, it may be more common than we realize and may be underdiagnosed due to lack of awareness. It is important to document as many cases of Axin2 mutations as possible to better understand a patient’s future risk of developing colon cancer; this will help to create guidelines and direct management for future physicians. Finally, as we begin to investigate molecular targets, such as KRAS and BRAF for therapeutic intervention, understanding the pathogenesis of Axin2 could make it a target as well.
